# Anomalous origin of the left coronary artery from the pulmonary artery presenting as dilated cardiomyopathy: a case report

**DOI:** 10.1186/1752-1947-8-170

**Published:** 2014-05-30

**Authors:** Rym Gribaa, Mehdi Slim, Helmi Ben Salem, Elyes Neffati, Essia Boughzela

**Affiliations:** 1 Department of Cardiology, Sahloul Hospital, Sousse, Tunisia

**Keywords:** Anomalous origin left coronary artery from the pulmonary artery, Imaging, Surgical treatment

## Abstract

**Introduction:**

Anomalous origin of the left coronary artery from the pulmonary artery is a rare congenital anomaly and one of the causes of myocardial ischemia. The usual clinical course is severe left-sided heart failure and mitral valve insufficiency presenting during the first months of life.

**Case presentation:**

We report the case of a 6-month-old Tunisian girl who presented with dilated cardiomyopathy. Echocardiography suspected anomalous origin of the left coronary artery. The definitive diagnosis of anomalous origin of the left coronary artery from the pulmonary artery was reached by multislice computed tomography and coronary angiography.

**Conclusion:**

In cases of dilated cardiomyopathy, anomalous origin of the left coronary artery from the pulmonary artery syndrome has to be kept in mind as a surgically correctable cause.

## Introduction

Anomalous origin of the left coronary artery from the pulmonary artery (ALCAPA) is a rare congenital anomaly occurring in 1 of 300,000 births [1]. As pulmonary artery (PA) pressure lowers to one-third of that of systemic pressure during the neonatal period, left coronary flow will tend to reverse into the PA resulting in coronary steal and a left to right shunt, with subsequent decreased myocardial perfusion and volume overload. When left untreated, it carries 90% mortality in the first year of life, largely from myocardial ischemia and heart failure [2]. In our case report, a 6-month-old girl presented with dilated cardiomyopathy (DCM) due to ALCAPA suspected by echocardiography and confirmed by computed tomography (CT) coronary angiography and coronary artery angiography. The report aims at emphasizing the importance of keeping this syndrome in mind in patients with DCM.

## Case presentation

**Figure 1:**
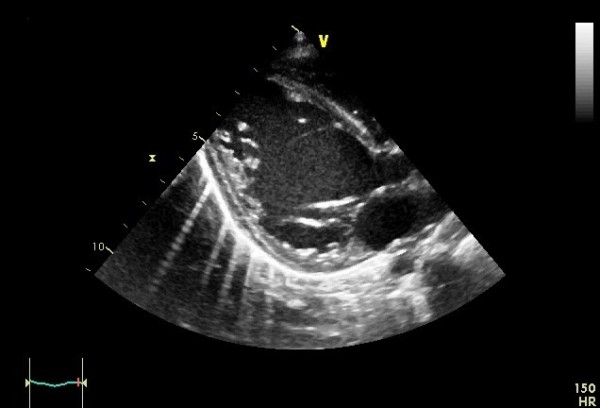
**Transthoracic echocardiography.** Dilated left ventricle.

**Figure 2:**
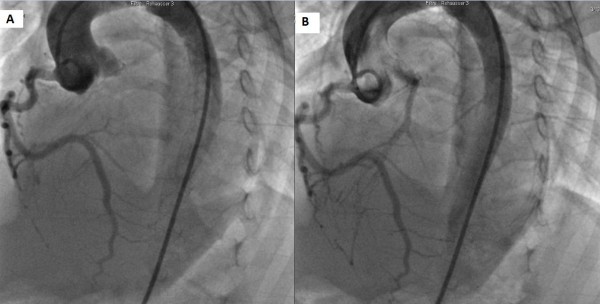
**Cineaortography.** Panel **(A)**: dilated right coronary artery, Panel **(B)**: retrograde filling of the left from the right coronary artery through collaterals.

**Figure 3:**
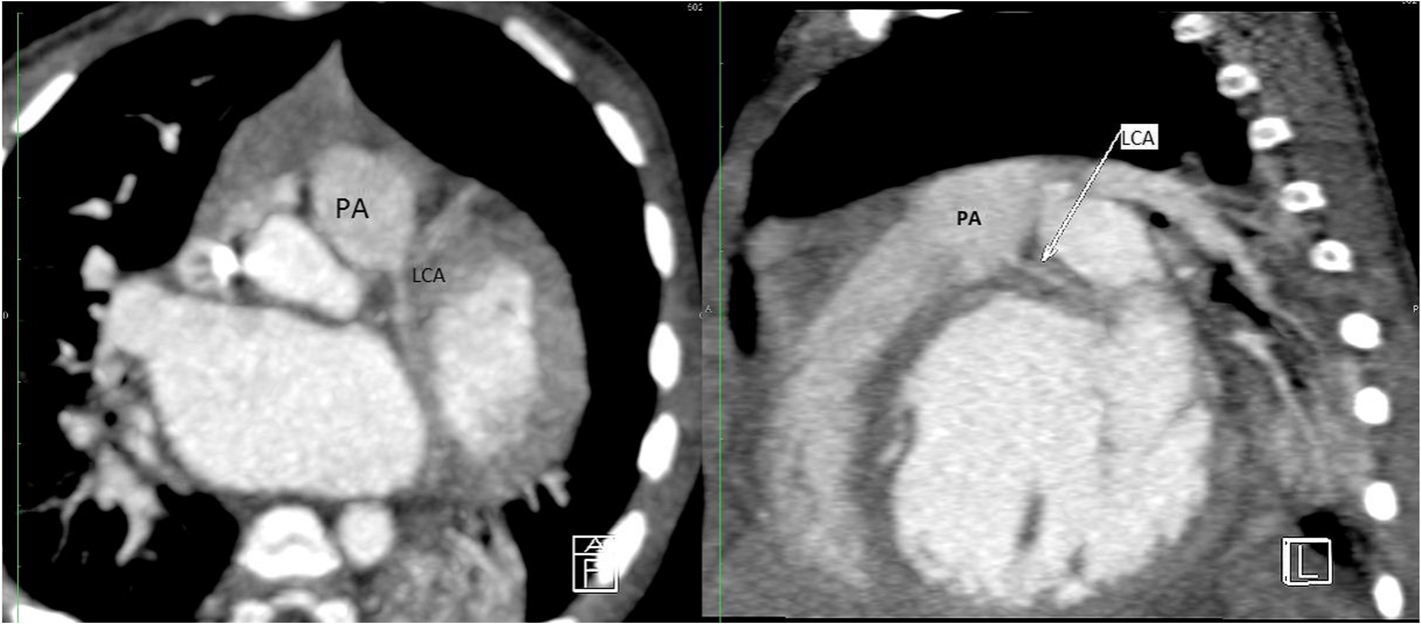
**Computed tomography angiography MultiPlan.** Left coronary artery emerging from the pulmonary artery trunk. LCA: Left coronary artery. PA: Pulmonary artery.

**Figure 4:**
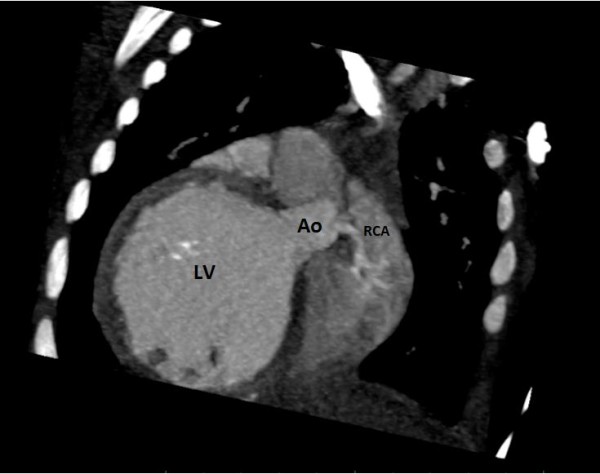
**Computed tomography angiography MultiPlan.** Right coronary artery emerging from aorta. Ao: Aorta. LV: Left ventricle. RCA: Right coronary artery.

A 6-month-old Tunisian girl presented with severe heart failure. Findings on physical examination were: weight, 6kg (−1, 3 standard deviation); length, 64cm (+0.2 standard deviation); heart rate, 120 beats/minute; and blood pressure, 100/60mmHg. There was no cardiac murmur. Electrocardiography (ECG) findings were left ventricular hypertrophy, abnormal Q waves in leads I, AVL, V5, and V6. In chest roentgen, her cardiothoracic index was 62%. Transthoracic echocardiography (TTE) showed a dilated and hypokinetic left ventricle with an ejection fraction (EF) of 35% and mild mitral regurgitation (Figure 1). Her right coronary artery (RCA) was dilated. The origin of her left coronary artery (LCA) was not seen making anomalous origin of her LCA strongly suspect. No associated cardiac anomalies were noted. The baby underwent cineaortography which showed an enlarged RCA arising from her aorta (Ao) and a retrograde filling of her LCA through collaterals from her RCA (Figure 2). ECG-gated CT-angiography, subsequently confirmed the diagnosis when revealing a RCA arising from her Ao with anomalous origin of her LCA emerging from her PA (Figures 3 and 4). Consequently, she underwent an uncomplicated aortic reimplantation of her left main coronary artery. No perioperative or postoperative complication occurred and she was discharged 10 days after surgery. She was on full dose of angiotensin-converting enzyme inhibitors and beta blockers.

She was still alive 6 months after surgery with a good functional status and normal growth. Echocardiographies performed during follow up found an improvement in systolic function with an EF of 45% with persisting mild mitral insufficiency.

## Discussion

ALCAPA syndrome was briefly described in 1866 [3] and again in 1908 [4]. However, the first clinical description was not published until 1933 by Bland, White and Garland [5], and the condition is also known as Bland-White-Garland syndrome. ALCAPA occurs approximately in 0.25 to 0.5% of children with congenital heart disease [6]. It is usually an isolated cardiac anomaly but, in 5% of the cases, has been described with coarctation of the Ao, atrial or ventricular septal defects [7]. During the fetal period, both systemic and pulmonary arterial pressures and oxygen saturation are equal. However, after birth, the PA contains desaturated blood at pressures that fall below systemic pressures. From the second month of life, as the vascular resistance drops in the pulmonary vascular bed, pulmonary pressure starts to decline as well. The ductus arteriosus closes and flow from the LCA reverses, which reduces the perfusion pressure of the ectopic LCA. The left ventricle is perfused with desaturated blood at low pressures. Myocardial ischemia may occur with resultant compromise of left ventricular function without a well-developed collateral circulation between the RCA and the LCA. In fact collateral circulation determines both the area of myocardial ischemia and whether symptoms will develop [8]. During infancy, ALCAPA syndrome can be fatal and patients present with myocardial infarction, left ventricular dysfunction, mitral regurgitation, or silent myocardial ischemia, which can lead to sudden cardiac death [9]. While adult patients can be completely asymptomatic, they may present with angina, dyspnea, syncope, myocardial infarction, arrhythmia or sudden cardiac death [6]. The electrocardiogram of a baby with ALCAPA usually shows typical signs of an anterolateral myocardial infarction, manifested by abnormal Q waves in leads I, AVL, V5, and V6, as well as by transient ST changes in these leads. The ECG may be also entirely within normal limits [10].

The diagnosis of coronary artery anomalies with the TTE is difficult because it may not always be possible to show the origins of the coronary arteries in adult patients. It is easier in newborns. In some cases, the anomalous origin of the left coronary system as well as the retrograde flow into the PA may be seen directly [11]. If there is a strong clinical or echocardiographic-based suspicion about the existence of this anomaly, then coronary or CT angiography should definitely be performed. In our case, the left ventricular dysfunction and failure to locate the LCA origin on TTE resulted in a high suspicion of anomalous origin of the LCA.

CT coronary angiography is a valuable noninvasive tool for showing such anomalous coronary arteries. This method also facilitates an accurate and detailed description of the origin and course of the coronary arteries and indicates a prognosis of the anomalous coronary arteries [12]. Although the diagnosis of ALCAPA is possible with noninvasive modalities, coronary angiography remains the gold standard method. The diagnosis is usually made when a dilated and tortuous RCA with collateral filling of the LCA system, and variable degrees of shunting to the PA is shown. The sensitivity of an angiography may be limited in the diagnosis of an anomalous coronary artery due to its invasive nature [13]. In our case the diagnosis of ALCAPA was strongly suspected by angiography and confirmed by CT scan. More than 90% of patients with ALCAPA will die within the first year of life without surgical intervention. Rarely, conservative medical therapy in asymptomatic elderly patients might be a reasonable alternative [6]. In patients with ALCAPA syndrome, even if the patient is asymptomatic, or when ventricular arrhythmia and significant left-to-right shunt or risk of death is not present, surgical treatment is suggested. In our case, the patient was operated as soon as the diagnosis was reached. The objective of surgical treatment is to restore a normal coronary circulation and improve myocardial perfusion. A few surgical procedures are suggested based on the localization of the abnormal coronary artery ostium. Surgical reimplantation or bypass of the left main coronary with left internal mammary artery graft was recommended. Takeuchi *et al.* defined another surgical technique, involving formation of a tunnel with intrapulmonary baffle [14]. In our case the LCA was directly reimplantated to the Ao. After surgery regular follow up is mandatory especially during the recovery period because of arrhythmia risk and sudden death. Early treatment may prevent irreversible myocardial damage with its subsequent complications.

## Conclusions

In babies with DCM, coronary artery anomalies should be considered a priority in establishing a diagnosis. CT angiography is a noninvasive tool for the diagnosis. Coronary reimplantation is the technique of choice for surgical correction. Early diagnosis and surgical treatment with optimal timing provide an excellent prognosis.

## Consent

Written informed consent was obtained from the patient’s legal guardian(s)/parent(s) for publication of this case report and any accompanying images. A copy of the written consent is available for review by the Editor-in-Chief of this journal.

## Abbreviations

ALCAPA: Anomalous origin of the left coronary artery from the pulmonary artery; Ao: Aorta; CT: Computed tomography; DCM: Dilated cardiomyopathy; ECG: Electrocardiography; EF: Ejection fraction; LCA: Left coronary artery; PA: Pulmonary artery; RCA: Right coronary artery; TTE: Transthoracic Echocardiography.

## Competing interests

The authors declare that they have no competing interests.

## Authors’ contributions

RG drafted the manuscript. MS contributed in collecting of data. HB contributed in writing the manuscript. EB contributed in correcting the manuscript. EB contributed in analysis, in interpretation of data, in the writing of the manuscript and in the decision to submit the manuscript for publication. All authors read and approved the final manuscript.

## Note

Research laboratory "Heart Failure" (LR12SP09).
